# Nurses’ Knowledge, Attitudes and Practices on the Management of *Clostridioides difficile* Infection: A Cross-Sectional Study

**DOI:** 10.3390/antibiotics12030529

**Published:** 2023-03-07

**Authors:** Dania Comparcini, Valentina Simonetti, Francesco Vladimiro Segala, Francesco Di Gennaro, Davide Fiore Bavaro, Maria Antonietta Pompeo, Annalisa Saracino, Giancarlo Cicolini

**Affiliations:** 1 CdL Infermieristica di Ancona, Facoltà Medicina e Chirurgia, Università Politecnica delle Marche (Univpm), 60121 Ancona, Italy; 2 Dipartimento di Medicina e Chirurgia, LUM “Giuseppe Degennaro”, 70121 Bari, Italy; 3 Department of Precision and Regenerative Medicine and Ionian Area—(DiMePRe-J), University of Bari “Aldo Moro”, 70121 Bari, Italy; 4 Local Health Service, ASL02 Abruzzo, 66100 Chieti, Italy

**Keywords:** *Clostridioides difficile*, nurses, cross-sectional survey, infection prevention and control, guidelines

## Abstract

*Clostridioides difficile* is, worldwide, the leading cause of hospital-acquired infection. Outbreaks are largely related to antibiotic exposure and contact contamination, but little is known about *C. difficle* infection (CDI) awareness in the nurse population. We conducted a cross-sectional survey to study Italian nurses, based on CDI guidelines. We recruited 200 nurses working in 14 Italian hospitals. Using a one-way analysis of variance of knowledge scores, female nurses (mean 9.67 (standard deviation ± 1.63), *p* = 0.03), and nurses with a higher level of university education (mean 9.79 (SD ± 1.67), *p* = 0.04) were demonstrated to have better knowledge about CDI. In addition, 92.5% (n = 184) of the sample declared that they did not have specific postgraduate training about CDI. Seventy-four percent (n = 149) of the respondents declared that they used procedures, protocols and guidelines about CDI in their workplace, but only 46.5% (n = 93) reported using *C. difficile*-specific bundles during their daily practice. In conclusion, our study highlights a lack of knowledge concerning CDI clinical guidelines among Italian nurses.

## 1. Introduction

*Clostridioides difficile* is an opportunistic, Gram-positive, anaerobic, spore-forming bacillus that can be widely found in the intestinal tract of humans and animals and in the environment [1]. In recent years, *C. difficile* has increasingly been reported as the most common pathogen causing healthcare-associated infection [2], the most common etiological agent of infective diarrhea in developed countries, and a leading cause of healthcare expenditure, with an estimated excess in healthcare costs of USD 11,285 per case in the United States alone [3].

In Europe and US, incidence rates of hospital-acquired *C. difficile* infection (CDI) range from eight to two cases per 100 patient days, showing a stable trend during the last decade [4]. However, the overall burden posed by this infection has led the Centers for Disease Control and Prevention to classify it among pathogens that represent an “Urgent Threat” to public health [5]. In the United States alone, CDI was responsible for an estimated of 250,000 clinical infections and was associated with up to 13,000 deaths in the year 2013. In European hospitals, 40,000 inpatients with CDI are potentially undiagnosed every year [6].

Incidence of *C. difficile* infection is strictly associated with antimicrobial exposure, since an antibiotic-driven shift in the composition of intestinal microbiota results in a loss to colonization resistance and, in the colonized host, antibiotic exposure possibly contributes to the pathogenesis of active infection through impairment of secondary bile acid production. In particular, the production of the primary bile acid taurocholate induces *C. difficile* by stimulating spore germination, and antimicrobial-induced depletions of gut bacteria might affect host capacity to convert primary into secondary bile acid, thus contributing to the development of active infection in a colonized host [7].

Almost all antibiotic classes have been associated with the infection, with penicillins, cephalosporins, clindamycin and fluoroquinolones carrying the highest risk [8]. Another major risk factor for the development of CDI is older age (≥65 years), with a relative risk up to 10 times higher than among younger patients [9]. Other known risk factors for CDI include hospitalization, gastrointestinal surgery [10], weakened immune system, organ transplantation, chemotherapy, inflammatory bowel disease [11], chronic kidney disease [12], environmental contamination [13], exposure to a known *C. difficile* carrier and having received a previous diagnosis of CDI, while the role of gastric acid suppression remains controversial [14]. 

Patient colonization rates range from 2.1% to 20%, with colonization risk increasing with longer hospital stays, prolonged antimicrobial exposure, close contact with people known to be infected or colonized [15] and in nursing home residents [16]. In hospitals and nursing homes, *C. difficile* viable spores can be found almost ubiquitously on toilets, telephones, healthcare workers’ hands, bedside furniture, stethoscopes and other medical devices, thus highlighting the key role played by nurses and healthcare workers (HCW) in complying with hand hygiene and other infection prevention and control (IPC) practices [17,18]. Indeed, along with reducing antibiotic prescription, effective strategies to reduce the overall burden of *C. difficile* infection include insolating the patient in a single room, wearing disposable gloves and gowns, washing hands with water and soap, daily decontamination of high-touch surfaces and full room decontamination after discharge. Thus, as key stakeholders in the correct implementation of infection control practices, nurses and other frontline healthcare workers play a critical role in reducing the burden of CDI in healthcare settings. Nurses are also involved in the care of patients with CDI, providing monitoring for signs and symptoms, education to both patients and relatives (including education on the proper use of antimicrobials) and coordination with other HCW as active participants in the antimicrobial stewardship team [19]. Therefore, effectiveness of any intervention in nosocomial settings is strictly reliant on the work of nurses and other healthcare workers. However, despite the amount of literature available on the topic, little is known about compliance with infection control practice guidelines among nurses, and no data are available from Italy, one of the countries most heavily affected by antimicrobial resistance [20]. The aim of this study is to assess knowledge, attitudes and practices about CDI among nurses working in southern Italy.

## 2. Results

Two hundred nurses working at 14 hospitals were enrolled in the study. Participants’ characteristics are summarized in Table 1. Overall, 75% (n = 150) of the respondents were female, 89% (n = 178) worked in medicine wards and 37% (n = 74) reported at least 16 years of working experience. Sixty-two percent of recruited nurses attained graduation, and almost half the nurses worked in the Local Health Service B.

**Figure 1:**
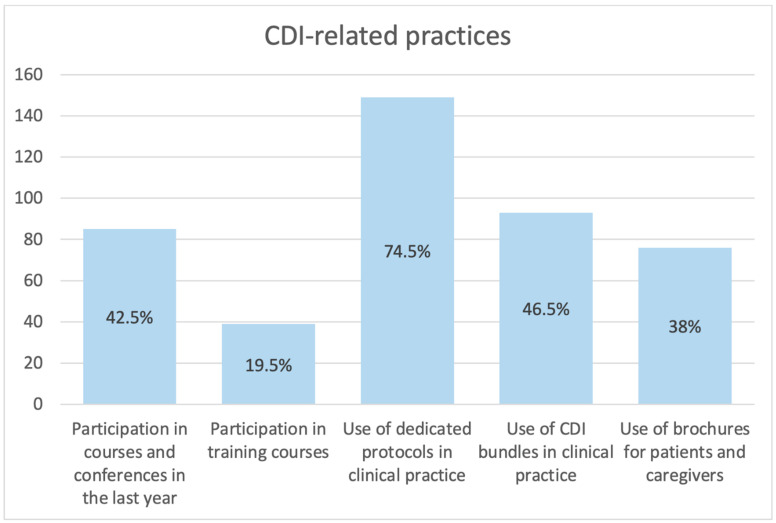
CDI-related practices. Histograms show the number of respondents who declared they had implemented the specific CDI-related practice on their daily professional activity. Percentages are reported in brackets.

In terms of attitudes and practices (Figure 1), most of the sample reported no specific postgraduate training about CDI (92.5%, n = 185). Of the respondents, 57.5% (n = 115) reported that they had not participated in any courses, conferences or meetings about CDI. Additionally, 157 respondents (78.5%) reported that no specific training courses were organized in their centers. On the other hand, 74.5% (n = 149) of the respondents reported that they routinely followed procedures, protocols and guidelines about CDI, but only 93 nurses (46.5%) reported that they adopted *C. difficile*-specific bundles during their working activity. In addition, only 76 (38%) respondents reported using brochures as an educational tool for patients and caregivers. 

In terms of knowledge, the vast majority of our respondents declared they knew what CDI is and 87% (n = 174) properly identified it as a bacterium. Likewise, 92% (n = 184) and 94% (n = 188) of the respondents correctly assessed, respectively, CDI clinical presentation and route of transmission.

**Figure 2:**
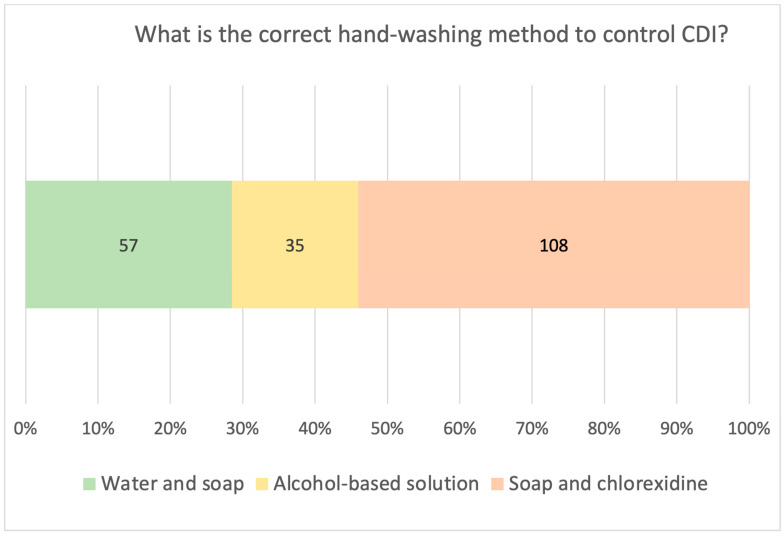
Knowledge of the proper hand washing method for CDI. The graph reports responses, in both percentages and numbers, to the question “What is the correct hand washing method to control CDI?”.

It is noteworthy that 23% (n = 49) of the respondents did not include gowns among required PPE in the case of CDI, 20% (n = 40) believed that CDI diagnosis requires a rectal swab and, as shown in Figure 2, only 57 (28.5%) nurses identified hand washing with water and soap as the proper IPC measure to be used in case of CDI. In addition, only 132 nurses (66%) knew that environmental disinfection should be carried out with sporicidal agents, and 62 nurses (31%) did not know that, in case of an outbreak, the proper management of patient clusters is to “cohort, isolate and use PPE“. Finally, only three-quarters of respondents (78%, n = 157) properly identified oral vancomycin as the first-line therapy against CDI.

The 15 knowledge items are shown in Table 2.

Analysis of variance of the CDI knowledge score showed that female nurses (mean 9.67; standard deviation ± 1.63) and nurses with a higher level of education—i.e.: graduated nurses (M 9.79; SD ± 1.67) and nurses with master’s degrees (M 9.5; SD ± 0.7)—demonstrated a significantly higher level of knowledge about CDI than, respectively, male nurses (9.06; SD ± 1.95; *p* = 0.03) and nurses who had attained only nursing university degrees (M 8.54; SD ± 2.15; *p* = 0.04). Furthermore, in this study, nurses working in long-term care wards had a higher mean knowledge score than nurses working in medicine wards (M 10.31; SD ± 1.42 vs. M 9.42; SD ± 1.74; *p* = 0.02). We also recorded a statistically significant difference between centers, with nurses working in Local Health Service (LHS) A and LHS-D scoring better (*p* = 0.001) than nurses working in LHS-B and LHS-C. Finally, no significant difference was recorded according to the years of job experience. In addition, in this study, having completed CDI-specific post graduate education did not influence knowledge score results (*p* = 0.267).

## 3. Discussion

In the last decades, CDI has emerged as a global health problem and, along with antibiotic exposure minimization, compliance with IPC practices is the cornerstone for prevention of *C. difficile* nosocomial outbreaks [21]. In this regard, it should be noted that, to be effective, IPC measures require specific awareness, such as being aware that alcohol-based sanitizers are not effective in removing *C. difficile* spores, whereas washing hands with water and soap is [22]. To our knowledge, despite being a substantial threat to public health and requiring active involvement of the nurse personnel, few studies have expressly targeted this population. However, available surveys [23,24,25] showed similar results in terms of awareness of transmission mechanisms, clinical picture, diagnosis and required IPC measures. When compared to a survey conducted in 2009 by Aroori et al., our population demonstrated higher knowledge of CDI clinical presentation (40% vs. 92%), but fewer respondents identified water and soap as the proper hand washing method to be used to curb the spread of CDI (38% vs. 28.5%). Knowledge about transmission dynamics was also higher, when compared to a study conducted by Finnimore et al. among nurses working in Australia, which reported that almost half of their population believed that *C. difficile* might be transmitted by aerosol. 

Of note, other studies were limited by a small sample size and high heterogeneity and none of them was conducted in Italy, one of the countries most affected by antibiotic over-prescription and misuse [26]. This is relevant, since the magnitude of the antimicrobial resistance crisis, the loss of adherence to IPC practices experienced during the COVID-19 pandemic [27], the ageing of the population and the emergence of antibiotic resistant [28] and hyper-virulent strains of *C. difficile* [29] are all factors that necessitate strengthening the surveillance on infection prevention and control practices compliance among nurses. In addition, the clinical impact of CDI disinformation among healthcare workers is not clear, but it is reasonable to believe that HCW knowledge about this disease has some impact on patients’ outcomes.

On the other hand, our population had good levels of basic knowledge about symptoms, route of transmission and risk factors for CDI, with more than half of the respondents being aware that old age, immunosuppression, a prolonged hospital stay and overuse of antibiotics are the main predisposing factors for the incidence of this disease.

It is noteworthy that only a third of nurses enrolled in our study were aware that hand washing with soap and water is the single most effective means of preventing transmission. This is consistent with other studies [23,24], and it could be due, in part, to the misunderstanding that hand cleansing with alcohol gel is insufficient to prevent the transmission. Additionally, only a minority of our population knew that effective interventions in preventing hospital outbreaks include daily disinfection with chlorine-based products on high-touch surfaces (including bed rails, furniture, sinks and floors), medical devices (blood pressure cuffs, stethoscopes, thermometers) and patients’ rooms [30]. However, most nurses enrolled in our survey knew that proper case isolation and adequate use of personal protective equipment, particularly gloves and gowns, are strongly recommended as an important precautionary measure in all guidelines. Likewise, most nurses also knew that vancomycin is the first choice for CDI treatment. 

Several limitations should be acknowledged. First, the sample size was small and limited to only one Italian region, thus limiting the generalizability of the results. Second, the results presented here do not consider the guidelines from the European Society of Clinical Microbiology and Infectious Diseases [31] which, however, do not provide specific new recommendation in terms of infection prevention and control practices. Third, respondent recruitment was obtained through convenience sampling, and no response rate information was collected. Fourth, although it was informed by literature review, full validation of the questionnaire was not carried out before its administration. Fifth, in the statistical analysis, correction for multiple comparisons was not performed. Sixth, the main outcome of this study was the result obtained from a score evaluating the responses included in the “Knowledge” section of the questionnaire, and thus it did not incorporate answers included in the “Prevention and Management” section. This choice was supported by the assumption that high levels of knowledge are a good proxy for both a positive attitude and good clinical practice. Finally, the survey presented here was conducted between 2019 and 2020, and nurses’ knowledge about CDI may have partly changed since then.

## 4. Materials and Methods

### 4.1. Study Design and Setting

This was an observational, multicenter, cross-sectional study carried out from August 2019 to March 2020 by administering an anonymous questionnaire to nurses working in Italy. The study was approved by the Local Health Service (LHS) and, prior to questionnaire administration, informed consent was obtained from all the study participants. LSH is a public body of the Italian public administration, responsible for the provision of health services in each territory, that operates within the framework of the Italian National Health Service. To guarantee confidentiality and anonymity, participants put the completed data collection tool in an envelope and placed it inside a special box. A convenience sample of nurses working in medicine wards and long-term care wards in 14 Italian hospitals located in one Italian region was recruited for this study. All hospitals included in the study were administered by four Local Health Services that, for the purpose of this work, were coded as LHS-A, LHS-B, LHS-C and LHS-D.

### 4.2. Questionnaire Development

We created and validated a 30-item questionnaire based on CDC (Centers for Disease Control and Prevention) guidelines entitled “Clinical Practice Guidelines for *Clostridium Difficile* Infection in Adults and Children” and Italian guidelines entitled “Prevenzione e controllo delle infezioni da *Clostridium difficile*” [32,33]. Only recommendations IA and IB were used for the questionnaire. The questionnaire was structured in the following six sections: personal information about participant (items 1–12); hospital organization on infectious risk (items 13–16); definition, symptoms and transmission of CDI (items 17–20); diagnosis of CDI (items 21–22); prevention and management of CDI (items 23–29); pharmacological treatment (item 30). As well as consultation of the clinical guidelines, each item of the questionnaire was informed by literature review, was composed only of close-ended questions and was thoroughly tested by the authors before the start of administration.

### 4.3. Statistical Analysis

A descriptive analysis was performed to define the distribution of demographic and other characteristics of the sample. Means and standard deviations were calculated for continuous variables, while categorical variables were reported through absolute numbers and percentages. A score to summarize knowledge results was calculated and used as an outcome. One point was given for each correct answer to the questions included in the Knowledge section. Therefore, minimum and maximum values of the knowledge score were, respectively, 0 and 15 points. An independent t-test was applied to compare groups for continuous variables, whilst analysis of variance (ANOVA) was performed to ascertain the presence of significant effects of variables on the total scores for each section. The statistical significance was set at *p* < 0.05. All statistical analyses were performed with SPSS software 20.0 (SPSS Inc., Chicago, IL, USA).

## 5. Conclusions

In our population of Italian nurses, we recorded substantial gaps in knowledge in terms of *C. difficile* infection, in particular about hand washing methods and environmental decontamination routines. Education of healthcare professionals is an important, and often forgotten, area in the fight against CDI and other healthcare-associated infections.

The involvement of nurses is crucial in reducing the burden of *Clostridioides difficile* infection in healthcare settings. Nurses are often the first point of contact for patients with CDI and are responsible for implementing infection prevention and control measures to prevent the spread of the infection. Nurses are also key figures in the care of patients with CDI, providing education, monitoring for signs of infection, and coordinating activity with other members of the healthcare team. They play a critical role in preventing the transmission of CDI by implementing standard and transmission-based precautions, such as hand hygiene and environmental cleaning, and by properly using personal protective equipment. 

To overcome the great public health challenge posed by CDI and, more broadly, antimicrobial resistance, scientific organizations and future research should prioritize this population. Further prospective data are needed to explore nurses’ knowledge about CDI in other settings, as well as the association between high levels of knowledge and main CDI-related clinical outcomes.

## Figures and Tables

**Table 1:** Demographic characteristics of the sample (n = 200).

Characteristic	Total Number (%)
Female	150 (75)
Local Health Service	
A	19 (9.5)
B	95 (47.5)
C	28 (14)
D	58 (29)
Ward type	
Long-term care	22 (11)
Medicine	178 (89)
Work experience (years)	
<1	28 (14)
1–5	44 (22)
6–10	27 (13.5)
11–14	27 (13.5)
16–20	23 (11.5)
>20	51 (25.5)
Educational level	
Nursing School Degree	42 (21)
Nursing University Degree	31 (15.5)
Graduate	125 (62)
Master’s Degree	2 (1)

**Table 2:** Answer distribution to questions included in the Knowledge section (total = 200). Correct answers are underlined.

Item	Total Number (%)
1. Have you heard of *C. difficile?*	
Yes	195 (97.5)
No	5 (2.5)
2. *C. difficile* is	
Virus	7 (3.5)
Bacterium	174 (87)
Mycobacterium	17 (8.5)
3. What are the main symptoms of CDI?	
Watery diarrhea and dyspnea	14 (7)
Fever, vomiting	2 (1)
Watery diarrhea and abdominal pain	184 (92)
4. What is the transmission route of CDI?	
Fecal-oral route and contact	188 (94)
Airborne	0 (0)
Airborne and contact	12 (6)
5. What are the main risk factors for CDI?	
Old age, improper use of antibiotics, immunosuppression	22 (11)
Genetic predisposition, old age, improper use of antibiotics, immunosuppression	7 (3.5)
Long hospitalization, old age, improper use of antibiotics, immunosuppression	171 (85.5)
6. Diagnosis of CDI requires the use of	
Rectal swab	40 (20)
Test tube for fecal sample	135 (67.5)
Test tube for fecal occult blood	25 (12.5)
7. When is stool sampling necessary for *Clostridium difficile*?	
If the patient has several episodes of diarrhea If the patient has foul-smelling formed stools If the patient has melena	186 (93) 12 (6) 2 (1)
8. What is the correct hand washing method to prevent CDI?	
Water and soap	57 (28.5)
Alcohol solution Soap with chlorhexidine	35 (17.5) 108 (54)
9. Environmental disinfection must be performed with	
Alcohol-based products	64 (32)
UV rays	4 (2)
Sporicidal agents	132 (66)
10. How often should high-touch surfaces in CDI patients’ rooms be disinfected?	
Every day	82 (41)
Several times during the day	32 (16)
Only when the room is cleared	86 (43)
11. What is the correct PPE to use with patients with CDI?	
Gloves and gowns	154 (77)
Gloves and mask	43 (21.5)
Gloves and overshoes	3 (1.5)
12. What are the precautions to consider in a suspected case of CDI?	
Use PPE	34 (17)
Isolate the patient and use PPE	165 (82.5)
Isolate the patient only if you have not started pharmacological therapy	1 (0.5)
13. How should you act if more cases of CDI occur?	
Cohort patients, isolate and use PPE	138 (69)
Use PPE and start pharmacological therapy	30 (15)
Isolate only patients with suspected CDI	32 (16)
14. When can isolation measures be discontinued in a patient being treated for *C. difficile* diarrhea?	
48 h after the last diarrheal discharge	31 (15.5)
After repeating the confirmation of healing test	166 (83)
After a week of isolation	3 (1.5)
15. What is the first choice for CDI treatment?	
Oral vancomycin	157 (78.5)
Oral metronidazole	12 (6)
Intravenous metronidazole	31 (15.5)

## Data Availability

The data presented in this study are available on request from the corresponding author.
